# Smoke Abatement

**Published:** 1906-02-24

**Authors:** 


### SMOKE ABATEMENT.

IN a paper on the abatement of the smoke nuisance in cities Sir Oliver Lodge [1] points out the harm resulting from the deposit upon vegetable and animal fabrics of the greasy and acid constituents of black fogs. These constituents are the products of imperfect combustion of coal. Such an imperfect combustion, in addition to discharging into the atmosphere a large quantity of deleterious bodies, involves an enormous waste of chemical substances which might, under more scientific regulation, be turned to useful account. For example, in an ordinary domestic grate an immense quantity of combustible and therefore useful, though impure, gas, is discharged uncombusted up the chimney, owing to the faulty nature of the stoking and of the grate. In addition to this invisible waste, there is constantly in operation a waste of the grosser particles of carbon which constitute smoke. Every one can realise the loss depending upon the waste of smoke, but the gaseous loss is more likely to be forgotten, because it cannot be seen.

The whole question, therefore, turns upon successful combustion. The problem of combustion may be considered under three headings: 1. The purification of the material to be used. 2. Proper means for effecting complete combustion under conditions easily regulated and unaccompanied by the production of dust and dirt. 3. The utilisation without waste of the heat derived from such combustion.

As regards the first point, the author observes that it is quite impossible to combust crude coal scientifically. That is to say, for scientific combustion the crude coal must be first separated by appropriate means into a gaseous portion and a solid portion. This last, of course, constitutes coke. This material, of the highest use for furnaces and smelting purposes, should be retained for these uses only; but such furnaces should not be permitted in towns. They should be established somewhere near the pit's mouth in order to secure the economy afforded by nearness to the source of supply. Under an ideal system the separation of the gas from the solid residue would also take place in the neighbourhood of the coal-pit; the cost of carrying coke would thus be reduced to a minimum, the gas being subsequently purified and sent by large conduits to the nearest town. Under such a scheme the sole combustion to be permitted in towns would be the economical combustion of a purified gas. A second alternative would be to convert the whole of the coal—except its ash—into gas by a judicious supply of air and steam. This gas, known as "water gas," or "producer gas," or "Mond gas," according to its precise method of preparation, would be cheap and plentiful, at the same time it would be rather more difficult to purify, and does not supply, bulk for bulk, as much heat as does ordinary coal gas. The author takes occasion to comment upon the commonly heard objection: "But we don't like gas fires." He observes that what the public has to put up with under present conditions is nothing more nor less than a gas fire, but that the fire is supplied by bad gas, badly made and badly burnt. As regards the second matter—namely the conditions necessary for perfect combustion—it is pointed out that the first essential for perfect combustion is the absence of cold surfaces to check the ignition of gases. It is well known that a flame can only be produced by a gas when that gas has reached its own flash-point of temperature; therefore, whenever a flame plays upon a cold surface it is immediately quenched at its point of contact with the cold surface. By this means, much of the gas produced in an ordinary fire is driven uncombusted up the chimney to contaminate the atmosphere and diminish the profit from the fuel used. For this reason it is clear that there is in ordinary domestic grates far too much iron.

The third point, that is to say, the utilisation of heat without waste, is more a commercial than a hygienic matter and does not command our attention directly. The moral pointed by the whole problem is given by the author as follows: "Do not allow crude combustion of coal in towns, but supply them all day long with cheap gas from a distance."

[1] Jour. of Royal Sanitary Institute, Feb. 1906.

